# The Unhappy Shoulder: A Conceptual Review of the Psychosomatics of Shoulder Pain

**DOI:** 10.3390/jcm11185490

**Published:** 2022-09-19

**Authors:** Matthias Vogel, Marius Binneböse, Hannah Wallis, Christoph H. Lohmann, Florian Junne, Alexander Berth, Christian Riediger

**Affiliations:** 1 Department of Psychosomatic Medicine and Psychotherapy, Otto-von Guericke-University of Magdeburg, 39120 Magdeburg, Germany; 2 Department of Orthopedic Surgery, Otto-von Guericke-University of Magdeburg, 39120 Magdeburg, Germany

**Keywords:** shoulder, impingement, chronic pain, functional hemispheric lateralization, psychosomatic, negative affect

## Abstract

Introduction: Chronic pain is a multifaceted disorder genuinely entangled with psychic and psychosomatic symptoms, which are typically involved in the processes of chronification. The impingement syndrome of the shoulder is no exception to this rule, but several studies have shown respective peculiarities among those with pain and impingement of the shoulder. Notably, chronic pain is a lateralized experience, and, similarly, its psychosomatic correlates may be attached to the hemispheres functionally. Aim: The present review therefore gives an overview of the respective findings, with regard not only to psychopathology, but also to personality factors and psychologic trauma, since the latter are reportedly associated with chronic pain. Moreover, we acknowledge symmetry as a possible pathogenic factor. Methods: This narrative review followed the current standards for conducting narrative studies. Based on prior findings, our research strategy included the relevance of psychotraumatologic and symmetrical aspects, as well as comorbidity. We retrieved the relevant literature reporting on the impact of psychopathology as well as personality features on shoulder pain, as published up to January 2022 from the Medline database (1966–2022). Study selecton: We included numerous studies, and considered the contextual relevance of studies referring to the neuropsychosomatics of chronic pain. Results: Pain-specific fears, depression, and anxiety are important predictors of shoulder pain, and the latter is generally overrepresented in those with trauma and PTSD. Moreover, associations of shoulder pain with psychological variables are stronger as regards surgical therapies as compared to conservative ones. This may point to a specific and possibly trauma-related vulnerability for perioperative maladaptation. Additionally, functional hemispheric lateralization may explain some of those results given that limb pain is a naturally lateralized experience. Not least, psychosocial risk factors are shared between shoulder pain and its physical comorbidities (e.g., hypertension), and the incapacitated state of the shoulder is a massive threat to the function of the human body as a whole. Conclusions: This review suggests the involvement of psychosomatic and psychotraumatologic factors in shoulder impingement-related chronic pain, but the inconclusiveness and heterogeneity of the literature in the field is possibly suggestive of other determinants such as laterality.

## 1. Introduction

The term impingement of the shoulder refers to a chronic and painful dysfunction of the shoulder causing pain at the elevation and internal rotation of the humerus. The prevalence of shoulder impingement syndrome (SIS) differs between age cohorts and was reported to range from 4.7–46.7% in terms of 1-year prevalence [1]. Similarly, McBeth [2] estimated that 20–33% of the general population were likely to report shoulder pain. Moreover, shoulder pain is among the leading causes of disability, and the third ranking condition of chronic pain. SIS is multifactorial and associated with a variety of disorders, e.g., diabetes, arterial hypertension, and thyroid disorders, as well as adiposity. In the general population, lesions of the rotator cuff (85%) and/or impingement syndromes [3] represent the most frequent causes of shoulder pain. SIS is a chronic, regional pain syndrome and based on the mechanical irritation of subacromial structures. The corresponding pain manifests mostly at the abduction of the limb from 70°–120° (i.e., the so-called “painful arc”, and clinical hallmark), at working overhead, and when lying on the affected side of the body [3]. The theory of impingement posits that mechanical conflicts between different structures of the joint would lead to shoulder pain, the second to third ranking musculoskeletal disorder [4]. Mostly, its symptomatic course develops in the fifth decade and the age peak lies between 40 and 60 years. Dependent on the precipitating factors, impingement syndromes are classified as primarily extrinsic, secondarily extrinsic, intrinsic, and inner impingement [5]. Glenohumeral osteoarthritis (secondarily extrinsic) and rotator cuff tear (intrinsic) are two common causes of shoulder pain, and the manifestation in the shoulder is the third ranking of osteoarthritis [6]. Regarding the subacromial impingement, ref. [7] a contact between the rotator cuff and the acromion causes damage to the rotator cuff, which may in turn confine the subacromial space. As a result, the mobility of the joint is compromised, even to the extent of total immobility as possibly suffered from in frozen shoulder. The structures mainly involved in the respective mechanical irritation are the supraspinatus tendon and the bursa subacromialis, causing irritation of the acromion and the coracoacromial ligamentum. Besides immobilization, conservative treatment is based on pain-relieving medication, physical therapy, as well as steroid injections. Even under such conservative therapeutic regimes, an operation (e.g., acromioplasty) takes place in 30% of the cases due to the lack of pain relief. Unfortunately, however, the operation does not always successfully [8] establish the desired relief of pain, either.

A similar paradox is known with respect to arthroplasty, especially of the knee, where 25% of patients tend to be unsatisfied with the results of the operation [9]. Among the explanations for this are the associations between osteoarthritis and psychopathology, esp. negative affect [10], personality [11], and trauma [12]. Besides negative affect, the psychologic suffering associated with chronic pain involves specific fears such as catastrophizing, which contribute to central sensitization and hyperalgesia, as well as to allodynia by means of heightened awareness directed to the sensation of pain. Negative affect is an umbrella term for anxiety and depression, and neuroticism reflects the tendency to experience aversive emotional states, especially negative affect [13]. Differently phrased, neuroticism resembles emotional lability and, thus, a source of complication when coping with illness. In addition, neuroticism generates unstable mood thus causing emotional lability, which is often expressed as a depressed or anxious mood, including worries about health-related issues. Negative affect is linked to chronic pain, e.g., of the knee [12], the back [14], and the shoulder [15]. Moreover, negative affect predicts worse outcomes and less satisfaction with therapies for chronic pain, such as arthroplasty [10], and is possibly a predisposition for posttraumatic stress disorder (PTSD), a connection described in the frame of the diathesis–stress model of PTSD [16], which may be of relevance for invasive therapies performed in people with posttraumatic symptoms. Negative affect was shown to rise in those with postoperative pain after total knee arthroplasty (TKA) and is deemed capable of inducing specific (i.e., pain-related) fears [12,17]. Moreover, as far as TKA is concerned, dissociative symptoms may qualify as negative affect in that they are possibly capable of inducing pain catastrophizing, thus ultimately increasing the perception of pain [12]. Dissociation is understood as posttraumatic symptomatology, and several dissociative symptoms [18] are required for the diagnosis of PTSD (i.e., amnesia, hypermnesia, derealization). Interestingly, with respect to chronic pain, PTSD itself conveys a heightened risk [19], possibly based on the activation of a clinical psychopathologic cascade initiated by negative affect and dissociation. Negative affect and neuroticism are also associated with a variety of physical illnesses, e.g., osteoarthritis [20], hypertension, and diabetes [21]. In addition to its associations with those disorders, negative affect may be a function of the right hemisphere [22]. Considering the lateralized manner in which peripheral pain is inherently organized, those anatomic underpinnings of peripheral pain possibly influence its associations with psychopathology.

A growing body of literature suggests psychosomatic and psychic comorbidity to modulate the subjective experience of shoulder pain, as well. Against this background, psychosomatic comorbidity complicates the adaptation to shoulder disorders, calling for complex and integrated approaches to their treatment. The present review gives an integrative overview of the respective findings that are suggestive of a link between the algofunction (i.e., the combined status regarding pain and function of the shoulder) related to frozen shoulder and psychiatric or psychosomatic syndromes, as well as the potential role of shoulder surgery as a crystallization point for posttraumatic psychopathology. Moreover, we strive to strengthen the hypothesis of the lateralization of psychosomatic correlates of pain.

## 2. Methods

The present review follows the recommendations for a narrative review [23]. Hence, we intended to depict not only the extent, but also the range and nature, of these studies, especially with respect to the extent to which the respective findings are integratable in a conceptual frame derived from research on other pain sites. In particular, we were interested in weighing the findings against the background of psychosomatic and psychotraumatologic concept formation. We searched Medline (1966–2022), as of January 2022. Our search strategy included the following terms mapped to the appropriate MeSH subject headings: (“childhood trauma” OR “PTSD” OR “dissociation” OR “amnesia” OR “derealisation” OR “depersonalization”) AND (“surgery” OR “postoperative maladaptation” OR “perioperative maladaptation” OR “depression and anxiety” OR “posttraumatic distress” OR “perioperative negative affectivity” OR “somatization”). The following terms were also included: (“comorbidity”, “personality”, “borderline”, and emotional lability). Additional terms used in the search were “lateralized”, “hemisphere”, and “contralateral”, in order to account for the fundamental principles of central pain processing.

To be included in the review, papers needed to measure or focus on specific dimensions of psychological influence on shoulder pain. Peer-reviewed journal papers were included if they were: written in English, involved human participants, and described a measure for psychological influence. Quantitative, qualitative, and mixed-method studies were included in order to consider different aspects of measuring psychological influence. In particular, we sought to identify psychosomatic and further etiologic concepts which are suitable for the integration of physical and psychic symptoms alike. Among those concepts is that of lateralization, referring to the central processing of pain that not only includes the activation of the contralateral hemisphere, but also the functional lateralization of mood, potentially causing syndromes such as anxiety to take side with contralateral peripheral pain for anatomic reasons.

## 3. Results

After duplicates were removed, a total of >499 citations were identified. After exclusion based on title/abstract and following assessment of eligibility based on full texts, we considered 48 studies eligible for this review. Our Medline search revealed a heterogenous set of endpoints and independent variables reflecting psychological disturbances. Although the hospital anxiety and depression scale was often reported, other measures of anxiety and depression were also deployed. In addition, specific fears such as pain catastrophizing and kinesiophobia are reported as predictors of the algofunction by three studies. Another important approach to the study question was the analysis of patient expectations as exemplified by Oh et al. [24]. Fewer studies reported personality characteristics as predictors of the algofunction in shoulder impingement. Table A1 (see Appendix A) gives an overview of those studies. As we hypothesized, the literature reviewed here was in line with the assumption of a posttraumatic pathway of maladaptation after shoulder surgery, and, in addition, the findings did not contradict the assumption of a lateralized pattern of association between shoulder pain and psychiatric syndromes. To make matters even more complex, central sensitization may not only involve lateralization, but also affect patterns of immunologic response. Thus, the present review could mark a new field of study with respect to the (psycho-)dynamics of shoulder pain.

## 4. Discussion

### 4.1. Psychosocial Correlates of Chronic Shoulder Pain

In clinical settings, the perception of patients with shoulder pain is often characterized by the impression of neurotic alignment, tenseness, and a lower pain threshold [25]. Moreover, the more peculiar the patient is in terms of the emotional presentation, the longer the duration, and the greater the severity of his or her disability was found to be [26]. Since shoulder impingement syndromes are among the top representatives of causes for chronic pain, the associations of chronic pain with psychosomatic features are likewise manifest in shoulder impingement, as well. Accordingly, Cho et al. [27] reported the preoperative association between depression and joint dysfunction as well as quality of life, all of them assessed preoperatively. Similarly, as for the prospective perspective, Dekker et al. [28] found higher scores on the hospital anxiety and depression scale connected to less postoperative satisfaction after 6 months. Park et al. [29] found early (up to 6 months) postoperative pain and the range of motion (RoM) affected by the preoperative presence of anxiety and depression. They concluded that psychological factors would delay the recovery as far as shoulder disorders are concerned. In addition, Cho et al. [30] showed the prediction of the joint function by preoperative depression scores, highlighting the functional influence of depression. Similarly, Martinez-Calderon et al. [31], in their review on psychosomatic influences on shoulder pain, report a relationship between depression, anxiety, emotional distress, and shoulder pain. On this note, depression and anxiety also predicted shoulder pain after 3 months in Debeer et al.’s [32] study, and this study showed improvement in psychological well-being to be linked to less physical pain, whereas the opposite, i.e., prediction of more pain by psychological deterioration, was not shown. This finding is interesting because it may point to a “somato-genic” nature of psychosocial findings in relation to shoulder pain and corresponds to the assumption of pain lateralization [22].

The fear avoidance model of chronic pain posits pain to be modulated by sensory amplification as a result of the attention drawn to its perception by specific fears, mostly pain catastrophizing. As a result, kinesiophobia, that is, the fear of motion and re-injury, rises and makes withdrawal from social and work-related contexts act as a contraphobic compromise, relieving from fear on the one hand, but leading to chronification on the other, especially as regards surgical treatments [12,33]. Less participation and activity, however, prompt less healthy lifestyles and increase the burden of pain. Accordingly, kinesiophobia predicted shoulder pain in Debeer et al.’s study [30], and, in addition, the authors report kinesiophobia to be more stable in men than in women over the study period. Likewise, Martinez-Calderon et al. [30] showed an association between preoperative concerns, fear avoidance, and chronic shoulder pain. In their recent review, DeBaets et al. [33] conclude fear avoidance to predict treatment outcomes only when the treatment was surgical, whereas, otherwise, outcome expectancies and self-efficacy predicted the respective outcomes. This finding begs the question of how much the operative setting poses a specific challenge for coping resources different from conservative settings. DeBaets et al.’s [33] conclusion might therefore be in line with the suggestion of a posttraumatic pathway of postoperative maladaptation after total knee arthroplasty that is hypothesized to result from prior traumatization, setting the stage for re-traumatization by the operation [12].

Moreover, Menendez et al. [34] found psychological factors (i.e., pain catastrophizing and insufficient coping) linked to shoulder pain, and report the same regarding social circumstances such as (un-)employment. In a prospective study, Thorpe et al. [35] identified a cluster of patients with surgery for rotator cuff repair, characterized by restricted psychological health, and whose pain and function of the shoulder were worse than in those without psychological problems. Potter et al. [36], however, found mild and moderate psychological distress not to correlate with the one-year outcomes of arthroscopic rotator cuff repair. Contrarily, Cho et al. [26] report a postoperative decrease in psychologic symptoms along with increased quality of life within a 12-month follow-up. Thus, as much as shoulder pain may induce depression, its reduction might act as an antidepressant. Counterintuitively, however, pain and injury of the dominant shoulder (limb) are associated with less hysteria and hypochondriasis [37]. The same authors highlight the relevance of the dominant upper limb for ambulation, body care, and movement [37]. As regards the impinged shoulder, this translates to dependency on others for daily activities and personal needs, shorter walking range, as well as less speed, and the authors underscore the finding of heightened hysteria as well as hypochondriasis in those with a lesion of the non-dominant limb, who are therefore considered to have a higher somatic awareness. However, this finding might reflect the more general fact that unilateral pain leads to the activation of the contralateral hemisphere. Ji et al. [38] have supposed a differential pattern of nociception in the left and right amygdala, and, in humans, the right hemispheric lateralization of amygdala function is linked to negative emotions [39]. Not least, shoulder disorders are heavily associated with problems returning to work, especially if coinciding with depression and anxiety. Thus, financial problems and disadvantageous prospects in the labor market often affect those with shoulder disorders disproportionately [40].

Considering the aforementioned involvement of specific fears and negative affect in the pathogenesis and maintenance of chronic pain, associations of shoulder pain with psychiatric disorders are to be expected. Accordingly, Bot et al. [41] found prevalences of depression (21%), anxiety (26%), schizophrenia (24%), as well as dementia (29%) elevated in candidates for shoulder arthroplasty. Apart from being, partly excessively, overrepresented, those entities were, except for schizophrenia, also linked to a higher risk of adverse events, including anemic states and longer institutionalized treatment for shoulder pain. Vice versa, depression increases the risk of rotator cuff tear and rotator cuff repair surgery remarkably [42]. That aside, psychiatric comorbidity is linked to increased cost and opioid use in relation to shoulder rotator cuff repair [43]. In addition to psychiatric disorders, sleep problems are present in 70–89% of the patients with rotator cuff tendinopathy [44]. Karels et al. [45] found somatization, kinesiophobia, and pain catastrophizing to predict the persistence of complaints over a 6-month follow-up, and classify their findings as corroborating the fear avoidance model. Similarly, Engebretsen et al. [46] report the association between pain-specific fears and shoulder pain, but [47] no significant effect of self-efficacy on either disability or on return to work. Several studies [48,49,50,51] report coping styles, in particular avoidant coping, to be associated with pain and disability of the shoulder.

The understanding of psychosomatic reactions to shoulder pain is connected to the question of causality, given that depression or anxiety could be a reaction to shoulder pain. This stance, however, is called into question by reports of non-linearity of the relationship between the extent of shoulder pain and depression [52]. As Badcock et al. [52] hypothesize, ceiling effects may preclude a further worsening impact of an increased load of depressive symptoms. Furthermore, these authors report the levels of disability to modulate those of depression, e.g., through problems sleeping. However, affective disorders and personality disorders are linked to arthritis, leading some authors to speculate about an essential relationship between those phenomena that, according to this stance, may be the symptoms of a single entity rather than representing different entities [24].

### 4.2. Recovery Expectancies

Another avenue of research investigates outcome expectancies in relation to the factual outcomes of therapy. On this note, Oh et al. [15] report outcome expectancies associated with the preoperative dysfunction of the joint, and Henn III et al. found preoperative positive expectations linked to more favorable postoperative results [53] after a 1 year follow-up. Likewise, Martinez-Calderon et al. [30] could show high levels of self-efficacy, resilience and expectations of recovery to be linked to levels of pain and disability albeit based on heterogenous studies involving different end-points and measures. Chester et al. [54], studying non-surgically managed shoulder pain, report that the prediction of pain (1/2 year) was best by the initial levels of shoulder pain, but strongly mediated by positive recovery expectations and optimism. Interestingly, the positive nature of the expectation may outweigh pain as a predictor. Accordingly, O’Malley et al. [55] found expectations to contribute to the short-term (3 months) functional outcome of shoulder disorders, explaining the interplay of functional improvements with functional expectations in terms of a specific capacity of negative expectations to undermine functional outcomes. In addition, Chester et al. [56] reported self-efficacy as a factor protective against shoulder pain. In addition, Henn III et al. [53], investigating a sample with primary surgical repair of a chronic rotator cuff tear, found positive expectations associated with the actual outcome with respect to function, even after controlling for a set of confounding variables including age, gender, smoking, workers’ compensation status, symptom duration, number of previous operations, number of comorbidities, tear size, and repair technique.

Notably, Bandura [57] understood expectations of self-efficacy as the extent to which an individual will strive to cope with a certain health condition. Therefore, expectations may be linked to more or less favorable ways of coping, prevailing mood, sickness behavior, and compliance, and thus impact the course of a disease effectively. That said, a patient´s motivation for treatment is largely guided by her or his expectancies. The motivation for treatment comprises a cognitive, as well as an affective, component [58], the latter being the subjective suffering and the secondary gain from illness, and the former referring to the disease-related concept. The individual connotation of these components may be inclined to more or less predominance of medical, psychic, or social factors, as far as symptoms and expected treatments are concerned. In osteoarthritis, illness perception is predictive of disability, especially the perception of the level of perceived control and consequences of osteoarthritis (OA) [16]. In turn, as disability progresses and the prognosis deteriorates, particularly the judgement of the individual affection with OA in terms of the number of symptoms, the belief about their negative impact, and chronicity, grows ever more pessimistic. Hence, poor illness perceptions seem to function as a self-fulfilling prophecy.

### 4.3. Higher-Order Factors: Temperament, Personality, and Posttraumatic Pathways

In that same sense, expectations reflect the tendencies that constitute the personality. For example, Basat et al. [59] found depressive temperament (operationalized as having withdrawn, the presence of self-blaming features, and the absence of steadiness) linked to a worse algofunctional outcome during a follow-up of almost 2 years. Likewise, Bru et al. [60] reported neuroticism and extraversion as well as trait anxiety correlated to pain ratings and concluded from their cross-sectional, retrospective study that personality traits would be more involved in shoulder pain than in back pain (p. 491), and Chiaramonte et al. report an association between primary adhesive capsulitis and perfectionism, novelty seeking (negative), and harm avoidance [61]. Not least, another study [62] found the effect of pain catastrophizing on joint function (though not pain) moderated by optimism.

With respect to such findings, however, Coronado et al. [62] also note the tendency of weakness that the associations between the outcomes of rotator cuff repair and psychological variables display, and the bias towards the study of surgical treatments in that respect as opposed to conservative therapies. Likewise, Sheikhzadeh et al. [63] in their formidable review have shown that psychosocial factors unfold their potential to predict pain in a more clear-cut manner with respect to surgical therapies as opposed to conservative ones. This result is in line with research highlighting the traumatic and interpersonal potential of surgery based on the suggestion that the violation of bodily integrity might bear the risk of re-traumatization for the traumatized [12].

Likewise, as to the link between chronic pain and psychological trauma, shoulder disorders are also overrepresented in veterans or (other) individuals with PTSD [61]. On this note, Wang et al. proposed a substantial overlap between pain, PTSD, and emotional factors, including strong feelings of anger, hatred, and aggression. Interestingly, some authors [33] note that the muscles of the back and the shoulder would be the first to react to tension [64].

### 4.4. How Are the Shoulder and the Psyche Connected?

The upper limb is especially characterized by dexterity, motion, and sensibility, all of which are at stake when the shoulder is functionally disabled and painful [65]. Mitchell et al. [66] suggest upper-limb injuries to compromise the function of the limb and, along with that, also to hamper psychosocial well-being. Surprisingly, however, as these authors further report, long-term outcomes are similar for those treated with amputation or limb salvage. Other authors find only a weak correlation and conclude there is no such thing as a “frozen shoulder personality” [31]. Notwithstanding, the comorbid conditions of diabetes and arterial hypertension are both linked to personality on genetic [67], clinical [68,69], and therapeutic [70] levels. Thus, the frequent coincidence of shoulder impingement, diabetes mellitus, and arterial hypertension may be rooted at least partly in shared psychosocial factors, which possibly promote their common manifestation [71]. For example, neuroticism is associated with higher levels of hypochondriasis, and may affect dietary habits and other aspects of illness behavior [72]. Generally, patients with the comorbidity of mental disorders and painful physical symptoms display higher levels of emotional distress, poorer physical functioning, and lower rates of help seeking [73].

As to the mechanisms linking psychiatric symptoms and shoulder pain, the elements of the fear avoidance model, precisely fear avoidance beliefs such as kinesiophobia and pain catastrophizing, are likely involved in the dynamics of the chronification of shoulder pain. Accordingly, there [30] was a relationship between emotional distress, depression, anxiety, preoperative concerns, and fear avoidance as well as chronic shoulder pain. However, these authors highlight the weakness of this association, as well as the presence of several biases. Moreover, the more widespread the pain, the more robust the association with psychological variables, leading the authors to conclude that generalized pain involving the shoulder is more bio-psycho-social than pain restricted to the shoulder only. Likewise, Sarquis et al. [74] found shoulder pain to be most disabling when embedded in a state of generalized pain, involving other sites as well. In line with this theorizing, knee pain especially predicts the spreading of pain [48]. For the sake of the full picture, it seems noteworthy that traditional and complementary methods (e.g., acupuncture) play only a minor role in the treatment of shoulder pain. Notwithstanding, integrative approaches are potentially promising as they effectively mitigate pain and improve states of negative affectivity, as well [75].

### 4.5. Comorbidity

Due to its complex anatomy, which involves not only the rotator cuff, but also capsulo-ligamentous structures as well as their chronic inflammation, fibrosis, and contracture, the shoulder unfolds a complicated and multifaceted etiopathology of its chronic affection with pain. Aspects of lifestyle, increasing load, immunological factors, as well as psychological features, not to mention hormonal and possibly genetic factors, all adjust the risk of SIS [76,77], and the profile of comorbidities of shoulder pain may serve to illustrate this stance. As regards the physical comorbidities, they apparently share some of the associative patterns between psychological factors and physical disease. Depression, anxiety, and neuroticism are overrepresented not only in those with shoulder pain, but also in those with arterial hypertension and diabetes [78].

One should nevertheless bear in mind that, while certain labilizing traits may have beneficial effects on one disorder, they may still be a deteriorating factor in another one [79,80].

Exemplifying this statement, neuroticism may confer a greater potential of health-related anxiety, promoting a healthier lifestyle and greater levels of adhesion with respect to cardiovascular disease [81]. Contrarily, neuroticism is not considered protective, but a vulnerability factor, when it comes to arthritis-related pain [82].

### 4.6. The Shoulder and the Knee: Key to Bipedalism

The knee is of even more importance and relevance for chronic pain than the shoulder. Reasons for the knee to bear complications include reduced participation, walking distance, and speed [83]. Yet, even more importantly, the knee is well-nigh key to bipedalism. The shoulder mirrors these qualities as it is also a peculiar joint with specific anatomy and function, is linked to chronic pain, and, once disordered, threatens the individual participation fundamentally. Both joints are essential underpinnings of the human two-legged mobility and our upright gait, and hence their pathologies have the potential of seriously crippling the body’s functional capabilities. This argument may help understand the psychological impact of those entities on general well-being. In addition, both entities are embedded in a pattern of physical morbidity that adds to the individual’s burden, hampering adaptive coping even more.

Not least [57], coping represents a competency depending on the individual’s psychic presentation, not only as far as mood disorders are concerned, but also with respect to the fundamental organization of the psyche as reflected in the individual’s personality. Thus, there may be a complex interaction between these dispositions and shoulder pain.

### 4.7. A Note on Centralized Pain and the Lateralized Nature of Pain and Its Psychosocial Correlates

In the nineteenth century, English physician John Spender elaborated a new classification of the initial symptoms of OA including changes in velocity and tension of the heart’s action, vasomotor changes, and specific neural symptoms [83]. The suggestion implied by this description was that the CNS could be functionally involved in the pathogenesis of OA. Contemporarily, this theory is revived insofar as there is the proposal of low-grade infection regulated top-down by a setpoint, as Morris et al. [84] state, that adjusts the neural, hormonal, inflammatory, and immune tone. The synovium and other joint structures are innervated by sympathetic and sensory fibers projecting to the thalamus and diencephalon. The higher the autonomic tone, the fewer anti-inflammatory effects of the parasympathicus are being brought to bear [84]. On the contrary, total knee arthroplasty is apparently associated with increased levels of circulating noradrenaline and adrenaline [85]. This high sympathetic tone leads to increased output of neutrophils and inflammatory monocytes from the bone marrow, cytokine production, and a heightened cell-mediated immune response [84].

Contemporarily, chronic pain is understood as centralized in the sense that pain would be intensified by central nervous processes and dysfunctions. Apart from pain, these processes often coincide with problems sleeping and memorizing, as well as fatigue, anxiety, or depression [86].

Independently from states of chronic pain, research suggests psychopathologic syndromes such as depression or anxiety to be functionally attached to the hemispheres, suggesting the psychosomatic epiphenomena of chronic pain to possibly be organized in a lateralized manner [32], not unlike the inherently lateralized pattern of limb pain. Hence, some of the repeatedly reported associations between lateralized chronic (shoulder) pain and psychopathology may reflect not only processes of sensitization, but also those of lateralization. Were this the case, then laterality might codetermine maladaptive patterns of adjustment based on differential left and right pathways of neural transmission [87]. Quite obviously, a purely peripheral concept of, e.g., osteoarthritis cannot explain the above outlined pattern of comorbidity. Moreover, centralized pain is less responsive to opioid treatments, further underscoring the clinical significance of this distinction. On the contrary, an unrecognized systematic pattern of association between lateralized pain and psychic as well as psychosomatic epiphenomena may contribute to the heterogeneity and inconclusiveness of the associations reported regarding, e.g., shoulder pain and psychopathology. On this note, a recent review weighed the significance of the reported associations by counting [63] the instances in which a psychological construct proved capable of the prediction of shoulder pain, or not. This illustrative and informative procedure may nevertheless omit the role of supra-ordinate factors such as laterality. In addition to other lateralized functions of the CNS, its neuroimmunomodulatory impact on the immune system may also indeed be a lateralized activity [88], with differences between the hemispheres pertaining to, e.g., the activation of macrophages involved in phagocytosis, inflammation, cytokine production, and antigen presentation, and to T cell activity affecting not only cellular, but also humoral, immunity. Moreover, with respect to traditional medicine there is the puzzling finding of beneficial effects of contralateral acupuncture on shoulder pain [75]. These are known to unfold on peripheral, spinal, and supraspinal levels [89,90], and they apparently rely on lateralization in terms of mirror symmetry, as well. Acupuncture is thus influential with respect to the antagonization of central sensitization, e.g., by means of segmental inhibition, or the activation of opioid or adrenergic receptors. The precise mechanism by which contralateral acupuncture is capable of mitigating limb pain, however, is unknown, but it likely involves neuroplastic processes of supraspinal origin [91], especially in connection with the anterior cingulate cortex [91]. Although promising, those alternative and complementary methods have nevertheless received only little attention in the literature on shoulder disorders.

## 5. Conclusions

The purpose of the present review was to summarize findings on the psychosomatics of shoulder pain, regarding psychopathologic syndromes and factors of the personality, and to connect them with specific psychosomatic, as well as psychotraumatologic, concept formation. As others did before, we underscore the frequent coincidence of psychopathology in relation to shoulder pain. Not unlike the knee, which—when painful—is robustly associated with a variety of mood and cognitive changes, and with higher-order factors such as neuroticism, shoulder pain is also entangled with psychopathology. A similar observation can be made regarding the physical comorbidities of shoulder pain, i.e., arterial hypertension and diabetes mellitus, which display a similar pattern of association with psychiatric syndromes. The present review demonstrates the complexity of psychosocial epiphenomena of chronic pain and of their interaction with shoulder pain, in particular, as well as the necessity of an integrated neuropsychosocial understanding and of a holistic treatment of pain.

## 6. Limitations

The present review summarizes the findings on the psychological and psychosomatic implications of shoulder pain. In doing so, it focusses on the interface between lateralized pain and the respective anatomy including the lateralization of pain afferents as well as the peculiarities of psychological trauma and its role in the chronification of pain. We discuss the included studies in accordance with these foci.

## Data Availability

Not applicable.

## Appendix A

**Table A1:** Characteristics of the included studies.

First Author (Ref.)	Year	No. of Partici-Pants	Mean Age (Years)	Duration of Symtpoms	Psychological Factor	Outcome Measure: Pain Intensity	Outcome Measure: Disability	Data Collection (Follow-Up)	Study Design
Badcock et al. [52]	2002	2606 (304 with unilateral shoulder pain) (142 completed the follow-up)	47.7	≥1 year to ≤3 years	Anxiety (HADS-A); depressive symptoms (HADS-D); emotional distress (HADS)	Pain intensity (5-point Likert scale)	Disability (disability questionnaire)	24 months	Longitudinal (prospective cohort study)
Bijsterbosch et al. [26]	2009	384 (241 completed all follow-ups)	59 (SD 7.5)	N/A	Illness perception (IPQ-R)	Pain intensity score modification of articular index for the assessment of osteoar-thritis	Disability (HAQ)	6 years	Longitudinal (prospective cohort study)
Bot et al. [41]	2014	348.842	69 (SD 15)	N/A	Diagnosis of depression, anxiety, schizophrenia, dementia	N/A	N/A	N/A	Retrospective cohort study
Cho et al. [30]	2015	58 (47 completed the follow-up)	57 (SD 8)	25 months (SD 36)	Anxiety (HADS-A); depressive symptoms (HADS-D); sleep disturbance (PSQI)	Pain intensity (VAS)	Disability (ASES)	12 months	Longitudinal (prospective cohort study)
Debeer et al. [32]	2021	72	53 (SD 7)	8 months	Anxiety and depressive symptoms (HADS), kinesophobia (TSK-11)	Pain intensity (VAS, SPADI)	Disability (SPADI)	3 months	Longitudinal (prospective cohort study)
Dekker et al. [28]	2016	86 (44 completed all follow-ups)	53.6 (depressed) 56.2 (non-depressed)	>3 months	Depressive symptoms (HADS)	Pain intensity (VAS, OSS)	Disability (OSS)	6 months	Longitudinal retrospective cohort study
Karels et al. [45]	2007	748 474 completed all follow-ups)	43.5 (SD 11.4)	≥3 months	Kinesophobia (TSK-11); depressive symptoms, anxiety, somatization, distress (4DSQ); catastrophizing (CSQ)	N/A	Disability (DASH)	6 months	Longitudinal (prospective cohort study)
Menendez et al. [34]	2015	139	58.1 (SD 14.3)	18.7 months (SD 26.8)	Depressive symptoms (PHQ-2); catastrophizing (PCS); self-efficacy (PSEQ)	Pain intensity (SPADI)	Disability (SPADI)	--	Cross-sectional cohort study
Oh et al. [15]	2012	174 (128 included)	58.8 (SD 8.2)	N/A	Preoperative expectations and concerns	Pain intensity (SF-36)	Disability (SST, Constant-Murley score)	Pre- and postoperative	Prospective cohort study
Potter et al. [36]	2015	269 (85 included)	62 (SD 2)	N/A	Distress (DRAM)	Pain intensity (VAS, ASES)	Disability (SST, ASES)	1 year	Prospective cohort study
Thorpe et al. [35]	2018	184 (124 completed all follow- ups)	54	N/A	Depressive symptoms and anxiety (DASS), catastrophizing (PCS); self-efficacy (PSEQ); kinesophobia (TSK-11)	Pain intensity (ASES)	Disability (ASES)	12 months	Longitudinal (prospective cohort study)
Koorevaar et al. [25]	2016	315	Not reported	40 (32)	Distress, depression, anxiety, and somatization	N/A	Function (DASH score)	12 months	Longitudinal (prospective cohort study)
Engebretsen et al. [47]	2010	104	48 (10.7)	N/A	HSCL-10, VAS	SPADI	SPADI	12 months	Prospective cohort study
Engebretsen et al. [46]	2015	200	49.8 (10.9)	N/A	Hopkins Symptom Check List	SPADI	SPADI	None	Cross-sectional cohort study
Wolfensberger et al. [49]	2016	158	47.1 (11.1)	5.5–15 months	HADS, PCS, TSK	DASH, brief pain inventory	DASH, patient Global Impression of Change measure	No follow-up	Retrospective
Badcock et al. [52]	2002	2606	47.7	N/A	HADS, PCS, TSK	VAS	N/A	2 years	Prospective cohort study
Henn III et al. [53]	2007	125	56.2 ± 11.4	16.0 ± 25.9 months	SF 36, MODEMS (partly)	DASH	DASH	1 year	Prospective, cross-sectional
Chester et al. [56]	2019	1030	57 (15.44)	N/A	Selected criteria	DASH	DASH	6 months	Prospective, cross-sectional
O’Malley et al. [55]	2004	199	51.6 (±15.7)	N/A	Patient Shoulder Expectancy Fulfillment, SF-12	FLEX-SF	FLEX-SF	3 months	Prospective, cross-sectional
Sarquis et al. [74]	2016	1410	20–59 years	N/A	SF-36, BSI-18	Questions about musculoskeletal pain	--	--	Prospective, cross-sectional
Zhang et al. [75]	2016	80	45.0 (7.4)		SF-36	DASH	DASH, Constant-Murley score	2 months	Prospective cohort study

ASES: American Shoulder and Elbow Surgeons (ASES) Society standardized shoulder assessment form; BSI: brief symptom inventory; CSQ: coping strategy questionnaire; DASH: disabilities of the arm, shoulder and hand questionnaire; DASS: depression anxiety stress scale; Disabilities of the Arm, Shoulder and Hand (DASH) questionnaire, DRAM: the Distress and Risk Assessment Method; FLEX-SF: Flexilevel Scale of Shoulder Function; HADS: hospital anxiety and depression rating scale; HAQ: health assessment questionnaire; HSCL: Hopkins Symptoms Check List; IPQ: illness perception questionnaire; MODEMS: Musculoskeletal Outcomes Data Evaluation and Management System; OSS: Oxford shoulder score; PCS: pain catastrophizing scale; PHQ: patient health questionnaire; PSEQ: pain self-efficacy questionnaire; PSQI: Pittsburgh sleep quality index; SF (−12/−36): short form (−12/−36) questionnaire; SPADI: Shoulder Pain and Disability Index, SST: simple shoulder test; TSK: Tampa scale of kinesiophobia; VAS: visual analogue scale; 4DSQ: the Four-Dimensional Symptom Questionnaire.
